# Antibody kinetics in primary- and secondary-care physicians with mild to moderate SARS-CoV-2 infection

**DOI:** 10.1080/22221751.2020.1793690

**Published:** 2020-07-20

**Authors:** Dorothea Orth-Höller, Angelika Eigentler, Lukas Weseslindtner, Johannes Möst

**Affiliations:** a MB-LAB – Clinical Microbiology Laboratory, Innsbruck, Austria; b Center of Virology, Medical University of Vienna, Vienna, Austria

**Keywords:** SARS-CoV-2, antibody kinetics, IgG, serological testing, primary- and secondary-care physicians

## Abstract

Three hundred and ninety-seven primary- and secondary-care physicians were tested for the presence of IgG (and IgA) antibodies against SARS-coronavirus-2 with a commercially available ELISA. In 19 of 20 individuals with PCR-proven infection and only mild to moderate symptoms not requiring hospitalization positive IgG levels occurred within two to three weeks. Among the remaining 377 persons without clear-cut evidence of infection, unequivocally positive IgG antibodies were found in only one, showing a surprisingly low prevalence (0.3%, 95% CI: 0.01–1.5) in physicians with likely contacts with infected patients in a region highly affected by the pandemic (Tyrol, Austria).

The severe acute respiratory syndrome coronavirus-2 (SARS-CoV-2), a novel human coronavirus (CoV), currently causes a massive pandemic and poses a major global health threat [1]. While PCR based assays are still the most sensitive and specific tool to identify infections, multiple SARS-CoV-2-specific antibody assays have only recently become available [2–4]. Serological tests bear the potential to complement PCR-based assays in the diagnosis, but distinctive antibody levels and kinetics, indicative for diverse infection stages, have to be evaluated for each available test system and related to a variable extent of disease severity [5–7].

In this study, we determined the prevalence of anti-SARS-CoV-2-IgG antibodies in primary- and secondary-care physicians, individuals with high level SARS-CoV-2 exposure, in a local region highly affected by the pandemic (Tyrol, Austria). We furthermore analysed antibody kinetics in a subgroup of physicians with PCR-confirmed infection, who comprehensively self-reported occurrence and severity of symptoms. In total, 560 primary- and secondary-care physicians were prospectively invited to provide a serum sample during a time period when the pandemic reached its preliminary peak (20th to 27th of March, 2020). Informed consent to participate for scientific purposes was obtained from all participants, who also completed a questionnaire, reporting the occurrence and severity of symptoms, results of eventually performed PCR tests and the mean number of daily patient contacts.

Out of the 560 contacted physicians, 397 finally participated in the study. Of those, 377 individuals (201 male, 176 female; median age: 51 years, median number of patient contacts 38 per day) had either a negative or no PCR test and only a minority reported symptoms compatible with a SARS-CoV-2 infection. In contrast, 20 individuals (15 male, 5 female; median age 55 years) displayed PCR confirmed infection, in 19 individuals with symptoms compatible with SARS-CoV-2 infection. In one physician (identified by contact-tracing) the infection remained completely asymptomatic. From those 20 infected individuals, 56 sequential serum samples were obtained during quarantine by a participating physician.

Serum samples were analysed for SARS-CoV-2-specific antibodies using Euroimmun SARS-CoV-2 IgG (and IgA) enzyme linked immunosorbent assay (ELISA) (Euroimmun, Lübeck, Germany). In samples from patients without PCR confirmed infection who displayed indeterminate or slightly positive IgG test results, we additionally performed the Wantai SARS-CoV-2 IgM and total antibody ELISAs (Beijing Wantai Biological Pharmacy Ent, Beijing, China). ELISAs were used as recommended by the manufacturers. Results by Euroimmun (IgG and IgA) and Wantai (IgM, total Abs) assays were classified as negative when antibody ratios were <0.8 or <0.9, respectively, positive with ratios >1.1 (and in between as indeterminate). All statistical analyses were performed using GraphPadPrism version 8.0. Wilson/Brown method was used to calculate 95% intervals of proportions (95% CI). Specificity of the tests, evaluated in 100 non-SARS-CoV-2 infected controls, was 83% and 98% for the Euroimmun IgA and IgG and 97% for the Wantai IgM and the Ab ELISAs, respectively.

Among the 377 physicians with unknown SARS-CoV-2-status, one individual without any symptoms since onset of the pandemic tested positive for SARS-CoV-2-specific IgG antibodies in two subsequent serum samples with high antibody levels (ratios >5, respectively). Eleven subjects displayed indeterminate or slightly positive IgG levels (ratios of 1.1–1.6), however, none of these individuals had an increase in IgG levels in subsequently acquired samples and the Wantai IgM and Ab ELISAs tested negative in all samples from those individuals. Interestingly, two individuals with IgG ratios between 1.0 and 1.4 showed positive IgA levels (ratios of 2.7 and 4.9). Since the Wantai IgM and Ab ELISAs also tested negative in these samples, all these 11 Euroimmun ELISA results were interpreted as unspecific, although occurrence of low-level IgG antibodies following an asymptomatic infection could not be completely excluded in these cases.

In the remaining 365 physicians, anti-SARS-CoV-2-IgG antibody tests revealed a negative result. Thus, 376 of 377 (99.7%) physicians with unknown SARS-CoV-2 status showed no clear serological evidence for SARS-CoV-2 infection.

However, among the 20 physicians with PCR confirmed SARS-CoV-2 infection, positive IgG levels were observed in 19 individuals, while one individual tested negative for IgG during the entire observational period until the 24th day after onset of disease. Thus, the Euroimmun IgG test showed a sensitivity of 95%, and the overall prevalence of IgG antibodies in the whole cohort of 397 physicians was 5.0% (95% CI: 3.3–7.7). Individual IgA and IgG antibody levels and kinetics are shown in Table 1 as well as in Supplementary Figure 1. Four of 14 tested individuals (29%) were IgG positive already at the second week after disease onset. At the third week after disease onset, 15 individuals (of 16 tested, 94%) developed positive IgG titers with high levels in most of the cases (ratios >4 in 11/16 participants at week 3, Table 1). Positive IgA-antibodies were detected in all individuals with PCR-confirmed SARS-CoV-2 infection, and in the majority of serum samples (47 out of 56). The study participant with absence of IgG antibodies showed a highly positive IgA-antibody level (ratio of 7.6) in week two after symptom onset.

**Table 1.** Antibody levels and kinetics in 20 PCR-confirmed SARS-CoV-2 infected patients.

	First week			Second week			Third week			Fourth week or later		
Patient	Day*	IgG	IgA	Day*	IgG	IgA	Day*	IgG	IgA	Day*	IgG	IgA
1	–	–	–	8	0.2	0.4	15	4.5	5.3	22	5.4	4.9
2	6	0.5	1.3	13	1.5	3.5	20	2.6	3.6	28	2.7	3.0
3	–	–	–	9	0.3	0.3	16	6.2	5.6	23	6.6	5.1
4	7	0.3	0.3	–	–	–	16	5.6	7.7	–	–	–
5	–	–	–	12	6.7	8.1	19	9.0	8.1	26	10.0	7.3
6	–	–	–	10	0.3	0.3	17	4.1	7.6	24	8.4	7.6
7	–	–	–	12	0.6	1.4	–	–	–	27/43	1.2/1.6	1.7/1.7
8	–	–	–	14	1.7	3.5	–	–	–	29/45	4.1/3.7	5.7/3.8
9	–	–	–	13	0.9	2.9	20	1.4	4.0	27	5.5	7.4
10	7	0.3	0.4	14	6.9	7.8	21	7.3	7.8	–	–	–
11	–	–	–	8	0.8	5.1	15	4.1	7.8	22	7.3	7.6
12	4	0.2	0.1	11	1.0	6.8	18	5.5	7.0	–	–	–
13	6	0.3	0.4	13	0.8	1.5	20	2.0	2.4	35	2.0	1.9
14	–	–	–	8	0.5	7.6	15	0.5	6.1	24	0.5	3.7
15	–	–	–	–	–	–	20	7.6	7.7	27	9.3	7.3
16	5	0.2	0.5	12	1.0	2.7	19	4.9	4.2	–	–	–
17	7	0.8	2.5	–	–	–	15	8.4	8.0	22	8.4	8.0
18	–	–	–	–	–	–	–	–	–	22/36	1.1/1.3	2.5/2.2
19	1	0.2	0.4	–	–	–	20	3.36	6.2	–	–	–
20	–	–	–	–	–	–	–	–	–	27	5.3	7.0

*time point of serum sampling in days after disease onset (for the one asymptomatic case after positive PCR).

Among the 19 physicians with symptomatic SARS-CoV-2 infection, the most commonly reported symptoms were fatigue, loss of olfactory sense, dry cough, muscle pain and headache (Suppl. Table 1). Four subjects reported only mild symptoms lasting not longer than 7 days, while 9 physicians reported symptoms lasting for 8–14 days, and in 6 physicians symptoms persisted for 15–21 days. Interestingly, the study participant with no detectable IgG seroconversion, displayed a very mild course of the disease (mild headache and conjunctivitis for 2–3 days). In this regard, SARS-CoV-2-specifc antibody levels have been proposed to correlate with the severity of the infection; however, previous reports mainly investigated antibody kinetics in hospitalized patients with severe to moderate courses [4,6,8,9].

In our prospective study with primary- and secondary-care physicians in a region heavily affected by the pandemic, we only found a low seroprevalence (1/377, 0.3%, 95% CI: 0.01–1.5) in participants with unknown SARS-CoV-2 status, a surprisingly low rate when considering that study participants had a high level of exposure due to frequent contacts with patients.

Nevertheless, in mild to moderately diseased physicians we demonstrate high agreement rates between SARS-CoV-2-PCR positive results and presence of specific IgA (100%) and IgG (95%) antibodies, which is in accordance with the recently reported detection rates in hospitalized patients, tested with the same ELISAs [10].

In our study, a significant increase and high detection rate of SARS-CoV-2-specific IgG until the third week after onset of symptoms was observed, while IgA already became detectable earlier (until 2nd week after symptom onset). These antibody kinetics should be considered when serological tests are used to aid PCR based assays in the diagnosis of SARS-CoV-2 infections.

## Supplementary Material

Letter__Emerg_Microbes_Infect_Antibody_kinetics_Orth_suppl.fig.1.docx

Letter__Emerg_Microbes_Infect_Antibody_kinetics_Orth_suppl.table1.docx
