# Supplementary figures and images for: Antibody kinetics in primary- and secondary-care physicians with mild to moderate SARS-CoV-2 infection
Source: Emerg Microbes Infect. 2020 Jul 20;9(1):1692–4. doi: 10.1080/22221751.2020.1793690 (PMC7473111; doi:10.1080/22221751.2020.1793690)

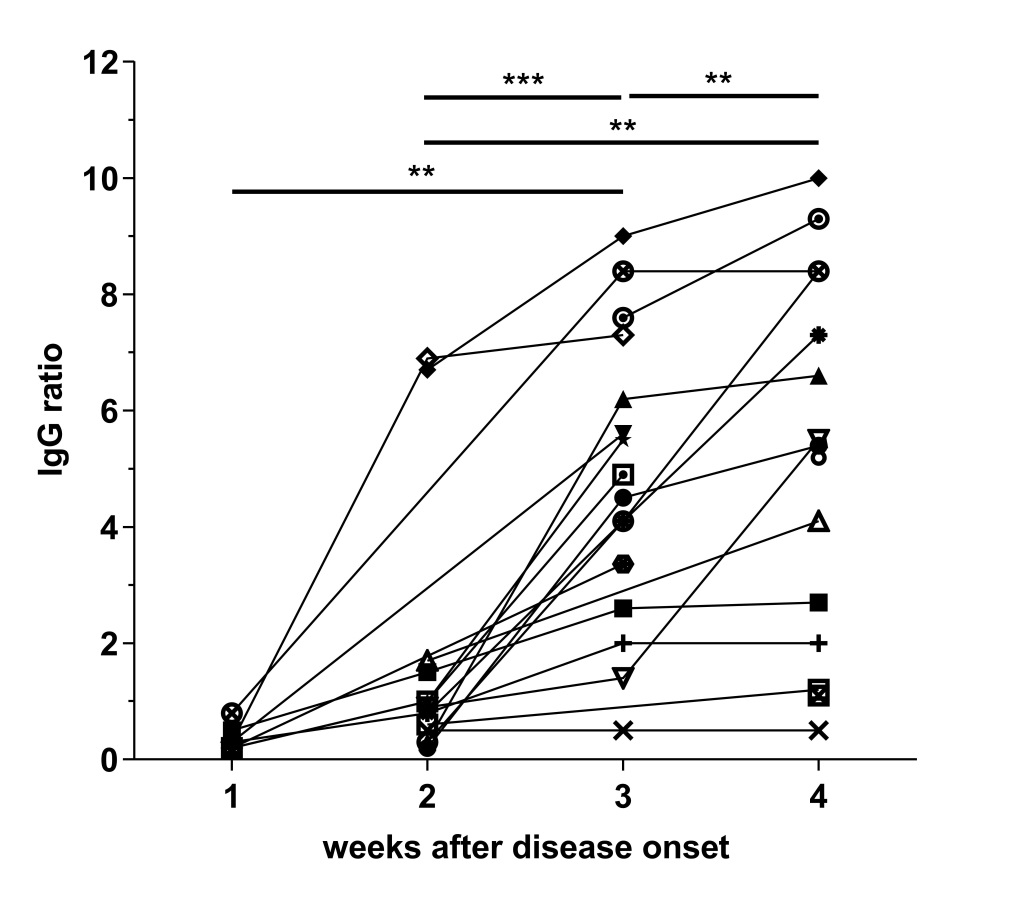
A)


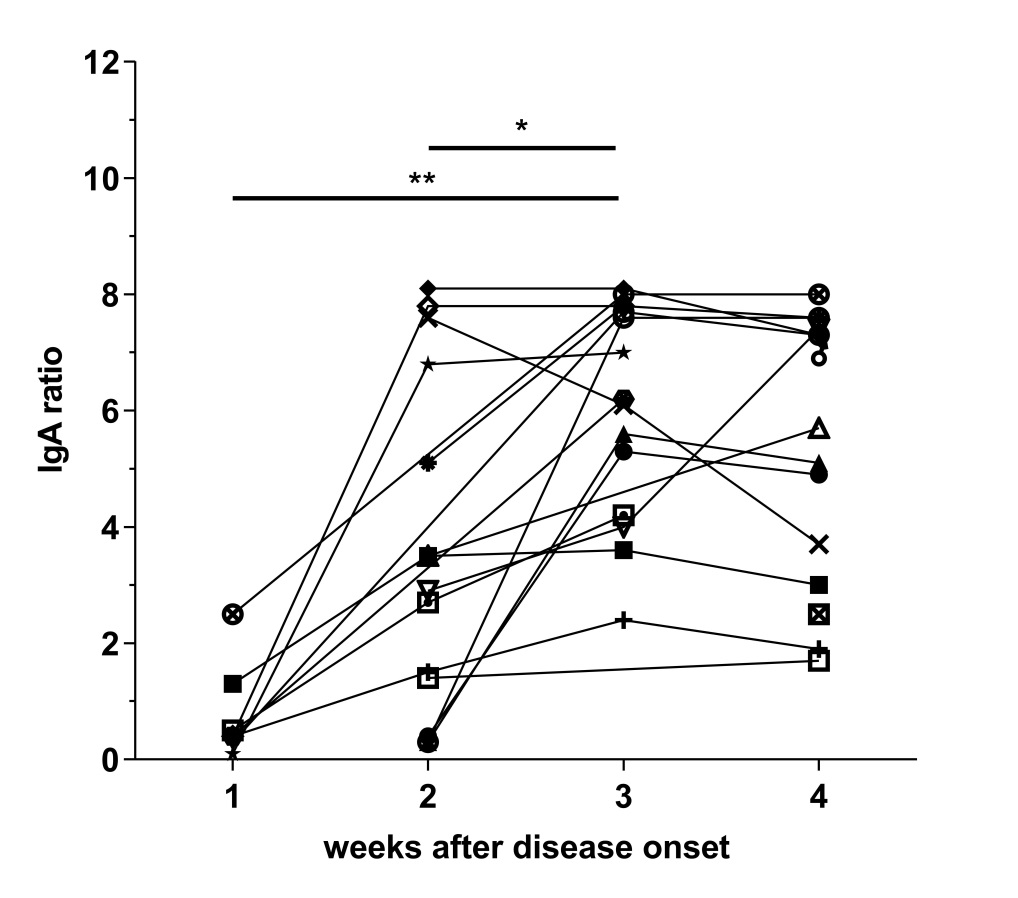
B)

Supplementary Figure 1 A,B

Supplement: Letter__Emerg_Microbes_Infect_Antibody_kinetics_Orth_suppl.fig.1.docx [file TEMI_A_1793690_SM4991.docx]
