# Supplementary material for: Antibody kinetics in primary- and secondary-care physicians with mild to moderate SARS-CoV-2 infection
Source: Emerg Microbes Infect. 2020 Jul 20;9(1):1692–4. doi: 10.1080/22221751.2020.1793690 (PMC7473111; doi:10.1080/22221751.2020.1793690)
Supplement: Letter__Emerg_Microbes_Infect_Antibody_kinetics_Orth_suppl.table1.docx [file TEMI_A_1793690_SM4990.docx]

Supplementary Table 1. Reported symptoms of the 19 SARS-CoV-2 infected and diseased individuals

| symptoms | severe (n=) | moderate (n=) | mild (n=) | absent (n=) |
| --- | --- | --- | --- | --- |
| fever | 0 | 4 | 7 | 8 |
| dry cough | 3 | 4 | 8 | 4 |
| productive cough | 1 | 0 | 4 | 14 |
| fatigue | 13 | 2 | 1 | 3 |
| loss of olfactory sense | 10 | 2 | 0 | 7 |
| dyspnoea | 2 | 1 | 5 | 11 |
| muscle pain | 8 | 3 | 3 | 5 |
| joint pain | 4 | 2 | 3 | 10 |
| headache | 5 | 7 | 4 | 3 |
| sore throat | 0 | 0 | 3 | 16 |
| nausea | 0 | 4 | 1 | 14 |
| vomiting | 0 | 0 | 0 | 19 |
| diarrhea | 1 | 3 | 6 | 9 |
